# Targeting the HMGB1-IL32 pathway to alleviate T cell exhaustion in epithelial ovarian cancer

**DOI:** 10.1007/s11357-025-01963-5

**Published:** 2025-11-15

**Authors:** Ankita Murmu, Balázs Győrffy

**Affiliations:** 1 Cancer Biomarker Research Group, Institute of Molecular Life Sciences, HUN-REN Research Centre for Natural Sciences, Magyar Tudósok Körútja 2, 1117 Budapest, Hungary; 2 Department of Bioinformatics, Semmelweis University, Tűzoltó U. 7-9, 1094 Budapest, Hungary; 3 Institute of Transdisciplinary Discoveries, Medical School, University of Pecs, 7624 Pecs, Hungary

**Keywords:** Immunotherapy, Pharmacology, Drug discovery, Targeted therapy, Epithelial ovarian cancer

## Abstract

Single-cell gene expression data can provide insights into cell–cell communication, enabling us to understand the interaction between cancer cells and microenvironmental cells. Here, our goal was to unravel how intercellular communication influences terminally exhausted CD8 + T cells in the ovarian tumor microenvironment. We processed and integrated ovarian cancer scRNA-Seq samples and delineated distinct cellular populations based on the expression patterns of established canonical marker genes. We performed a pseudotime trajectory analysis of CD8 + T cells and analyzed the communication of ovarian cancer cells with terminally exhausted CD8 + T cells. Investigating cell lineage and inferring pseudotimes revealed the transition of the CD8 + T cells from naïve-like to six different end-states, with central memory (35%), effector memory (31%), and terminally exhausted (25%) CD8 + T cells being the most abundant CD8 + T cell subtypes. Cell–cell communication analysis identified the *HMGB1*-*HAVCR2* ligand-receptor pair mediating communication from ovarian cancer cells to terminally exhausted CD8 + T cells. High Mobility Group Box 1 (*HMGB1*) was identified as a key ligand expressed in ovarian cancer cells influencing the *IL32* expression in terminally exhausted CD8 + T cells. The signaling path from *HMGB1* to *IL32* revealed *NFKB1* as the most significant signaling mediator and *TP53* as the most significant transcriptional regulator via which *HMGB1* influenced *IL32* expression in CD8 + T cells. The *HMGB1-IL32* signaling pathway identified in our analysis can serve as a therapy target for a new generation of adjuvant therapy designed to suppress and disrupt tumor cells’ influence on the microenvironment and enhance immunotherapy efficiency.

**Graphical Abstract:**

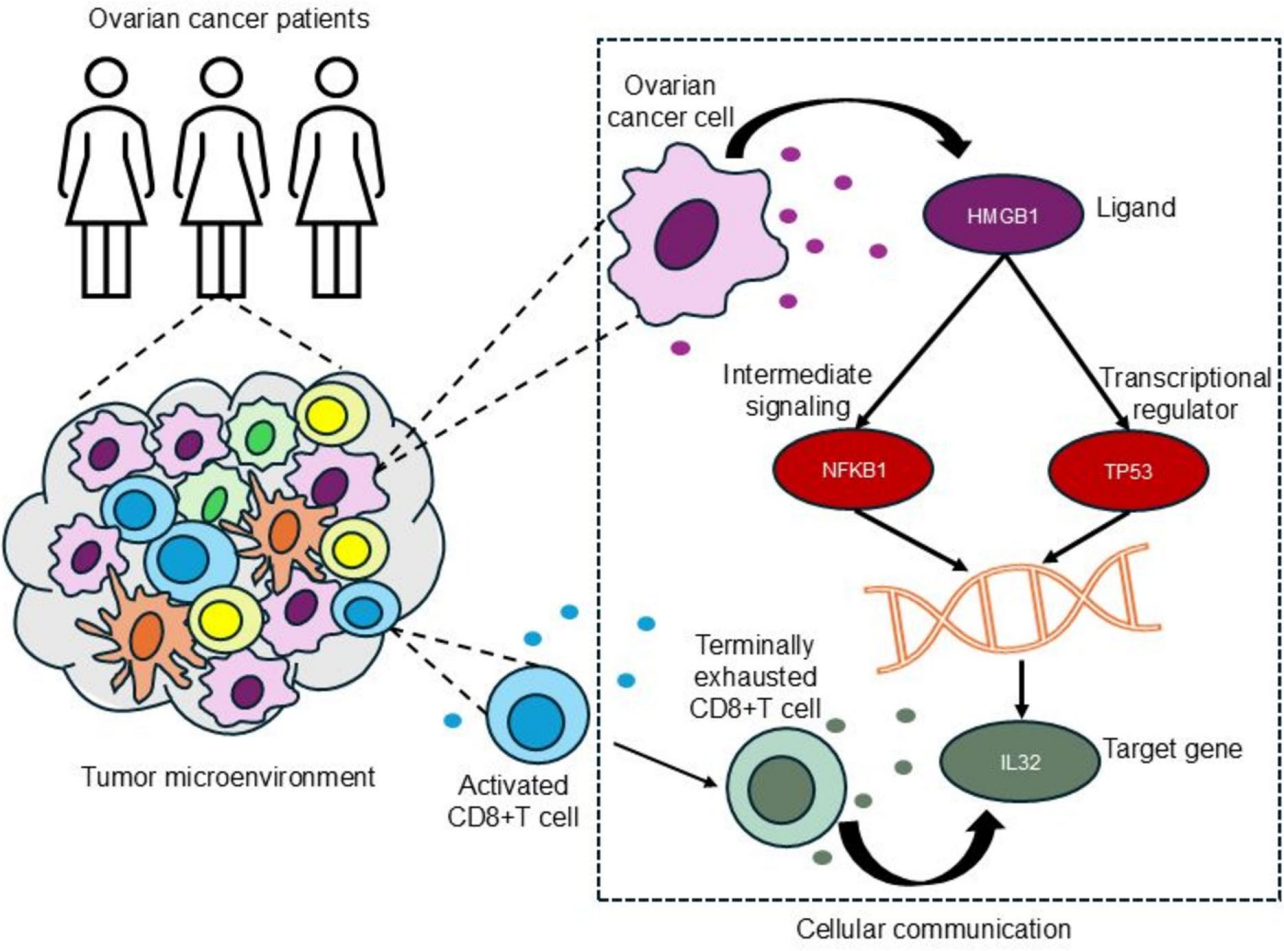

**Supplementary Information:**

The online version contains supplementary material available at 10.1007/s11357-025-01963-5.

## Introduction

Epithelial ovarian cancer represents a diverse group of malignancies with different histological subtypes [1]. These histological subtypes include endometrioid, clear cell, mucinous, and low-grade serous subtypes, with high-grade serous being the most prevalent subtype, accounting for a significant portion of ovarian cancer cases [2]. It was projected in 2020 that around 307,000 deaths will occur globally by 2040 [3]. These could be largely due to delayed diagnosis at an advanced stage leading to poor prognosis in the majority of women [4].

Surgery followed by chemotherapy has been the standard front-line treatment for advanced-stage ovarian cancer patients. In addition, PARP or poly (ADP-ribose) polymerase inhibitors are now available for front-line settings with chemotherapy [5]. However, a significant risk of recurrence has been reported in ovarian cancer patients [6]. New treatment strategies are of great need for these patients, and immunotherapy, including interventions like immune checkpoint blockade, cancer vaccines, and cell-based therapies, has been the subject of significant interest [7].

In this regard, the tumor microenvironment could be explored for therapeutic approaches. Tumor-infiltrating lymphocytes such as T cells, B cells, macrophages, or natural killer cells have been known to exist in the tumor microenvironment [8]. These diverse cell types influencing tumor progression and therapeutic response are considered promising targets [9]. Among them, T cells are the driving force of adaptive immunity, and CD8 + T cells are the major players in eliminating cancer cells. The presence of CD8 + T cells correlates with a favorable prognosis in ovarian cancer patients [10, 11].

Inducing an anti-tumor response of T cells is crucial for effective immunotherapy. However, tumor-infiltrating CD8 + T cells are unable to eliminate cancer cells from time to time as they can reach an exhausted or dysfunctional state throughout tumorigenesis [12]. In addition, cells in the tumor microenvironment transmit information for cellular activities. Cellular interactions of neighboring or long-distance cells happen through molecules like cytokines, chemokines, growth factors, adhesion molecules, as well as gap junctions. Communication between the malignant cells and normal cells was described to be mediated by various types of ligand-receptor interactions [13]. Studying cancer-microenvironment interactions could reveal potential diagnostic and therapeutic targets [14, 15]. As a result, understanding the interplay between tumors and the immune system in ovarian cancer patients could provide new avenues for improved treatment outcomes.

The advancement of single-cell technologies provides the opportunity for analyzing the intricacies of the tumor microenvironment. This enables the exploration of intercellular communication within the tumor microenvironment after identifying distinct cell populations. While previous studies have focused on analyzing ligand-receptor interactions between ovarian cancer cells and stromal or immune cells [16, 17], understanding how ovarian cancer cells drive exhaustion through ligand-induced transcriptional changes in target genes is crucial.

**Fig. 1:**
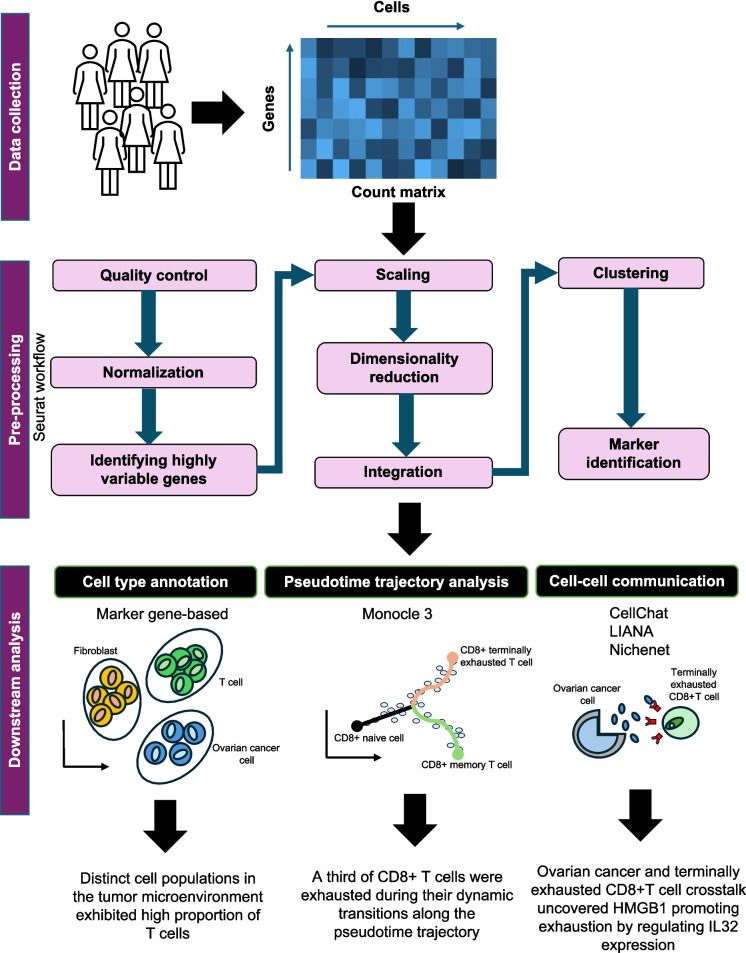
Overview of the study design shows data collection and pre-processing of ovarian cancer datasets using the Seurat package. The downstream analysis shows the marker gene-based cell type annotation, pseudotime trajectory analysis of CD8 + T cell subtypes, and cellular communication analysis from ovarian cancer cells to terminally exhausted CD8 + T cells

In this study, we integrated and characterized distinct cell types to scrutinize ovarian cancer using single-cell RNA-seq datasets. The developmental trajectory of CD8 + T cells with different end states was examined by pseudotime trajectory analysis. Cellular communication revealed key ligands expressed in ovarian cancer cells influencing and regulating gene expression in terminally exhausted CD8 + T cells. Signaling network analysis revealed signaling mediators and transcriptional regulators involved in signaling from ligand to target gene. The overview of the study is illustrated in Fig. 1. Together, this work unravels a key transcriptional modulator which could serve as a therapeutic target for ovarian cancer immunotherapy.

## Materials and methods

### Data collection

We searched the National Center for Biotechnology Information database for ovarian cancer Gene Expression Omnibus (GEO) datasets using the query “ovarian cancer” AND “single-cell RNA-Seq”. The results were filtered based on “organism”, “entry type”, and “study type”. Four different studies that consisted of 10X genomics datasets were selected. These datasets consisted of samples derived from ovarian and omentum tumor tissues. Raw data consisting of matrix, barcode, and feature files was obtained for each ovarian cancer patient. Dataset information with its accession numbers is provided in Table 1.

**Table 1:** Accession numbers of the samples used in the study

GEO accession	Sample accession	PubMed ID
GSE217517	GSM6720925	37864274 [62]
	GSM6720926	
	GSM6720927	
	GSM6720928	
	GSM6720929	
	GSM6720931	
	GSM6720932	
GSE173682	GSM5276939	34739872 [63]
	GSM5276940	
	GSM5276941	
	GSM5276943	
GSE154600	GSM4675273	32747365 [64]
	GSM4675274	
	GSM4675275	
	GSM4675276	
	GSM4675277	
GSE184880	GSM5599225	35675036 [65]
	GSM5599226	
	GSM5599227	
	GSM5599228	
	GSM5599229	
	GSM5599230	
	GSM5599231	

### Establishing the integrated database

All the data pre-processing steps were conducted using Seurat version 4.0.3 [18] in R version 4.4.2. For quality control, cells with a gene count of less than 300 and genes expressed in less than three cells were excluded for each dataset. Single cells with over 6,000 genes were filtered out to remove potential doublets. The percentage of mitochondrial genes for each sample was calculated using the *PercentageFeatureSet* function. To remove dying cells, cells with a mitochondrial reads percentage of more than 15% were excluded.

The datasets were log-transformed, and the 2000 most variable genes were selected using the *FindVariableFeatures* function. After selecting repeated variable features and identifying anchors, data integration was performed by canonical correlation analysis using *IntegrateData* to remove batch effects.

### Principal component analysis and clustering

The integrated data was linearly transformed using *ScaleData*. Principal component analysis (PCA) using *RunPCA* was the first technique applied to the scaled data for dimensional reduction. Then, Uniform Manifold Approximation and Projection (UMAP) reduction was performed on PCA with 25 dimensions, and the clusters were visualized. For graph-based clustering, the shared nearest neighbor method was used. The k-nearest neighbors of each cell were calculated by *FindNeighbors*, and cell clusters were formed based on the Louvain algorithm for a range of resolutions from 0.1 to 0.8.

### Identifying cell types

For annotating major cell types, we identified positive marker genes or differentially expressed genes between each cluster and the other cells using *FindAllMarkers* in Seurat to perform a Wilcoxon rank sum test with parameters logfc.threshold = 0.25, min.pct = 0.25. The cell type annotation was further validated based on marker genes used in previous studies (Table 2). We identified eleven major cell types. Then, we extracted the T cells from our annotated dataset to identify the T cell subtypes using ProjectTILs version 3.3.0 [19]. To obtain CD8+ T cell subtypes, our query T cells were first filtered and projected on the ProjectTILs human CD8+ TIL reference atlas that consists of 11,021 single-cell transcriptomes from 7 cancer types, including ovarian cancer. We further pre-processed and re-clustered the new CD8+ T cell subset at resolution 0.5 using Seurat.

**Table 2:** Marker genes from previous studies used for cell type annotation

Cell type	Reference (PubMed ID)	Suggested marker genes
T cell	35675036 [65]	CD3D
	36517593 [66]	CD2, CD3D, CCL5
	37046345 [67]	CD3D
B cell	36517593 [66]	CD79A, BANK1, MS4A1
	37046345 [67]	CD79A
Plasma cell	36517593 [66]	IGLC3, IGLC2, IGKC, CD79A
	35675036 [65]	CD79A
Endothelial cell	36517593 [66]	PECAM1, VWF, CLDN5
	35675036 [65]	PECAM1
	32747365 [64]	PECAM1
	37046345 [67]	PECMA1, VWF
Fibroblast	36517593 [66]	COL1A1, DCN, ACTA2, IGFBP7
	37046345 [67]	COL1A1, DCN
Smooth muscle	37488671 [68]	RGS5
Myeloid cell	36517593 [66]	LYZ, C1QA, C1QB, APOE
Natural killer cell	36517593 [66]	GNLY, GZMB
Cycling T/NK	36517593 [66]	CENPF, MKI67, TOP2A, HMGB2
	36973297 [69]	TOP2A, MKI67
Ovarian cancer cell	36517593 [66]	KRT18, CD24, KRT19, EPCAM, WFDC2
	34739872 [63]	WFDC2
Dendritic cell	36517593 [66]	IRF4, IRF8, PLAC8

### Trajectory analysis

For trajectory analysis, the Seurat object with CD8 + T cell subtypes was converted to a Monocle3 object, and data size factors were calculated. Monocle3 [20] version 1.3.4 was used for trajectory analysis of the CD8 + T cells. A single partition was assigned to all cells, and the *learn_graph* function was used to learn the trajectory graph. The naive cells were chosen as the “root” of the trajectory to allocate the cells in pseudotime. For each cell, a pseudotime value was assigned based on its position in the trajectory.

### Cell–cell communication analysis

We extracted ovarian cancer cells and the CD8 + T cell subtypes identified with ProjectTILs, and performed cell–cell communication analysis using CellChat version 2.1.1 [21]. CellChat identifies over-expressed ligands or receptors for each cell group. We used the CellChat database to include pathways and ligand-receptor interactions from the “Secreted Signaling” and “Extracellular Matrix Receptor (ECM)” categories. Based on the average expression values of ligands in one cell group and receptors in another cell group, a probability value less than 0.05 is produced for the interactions modeled by the law of mass action. To produce fewer but stronger interactions, we used the statistically robust mean method called “trimean” for calculating the average gene expression per cell group.

To assess the consistency of our CellChat results, we used LIANA version 0.1.14 [22] for ligand-receptor analysis. LIANA uses multiple resources and methods to aid in the investigation of cell–cell communication. For our analysis, we used the default consensus, which is based on five previously established cell–cell communication methods: Network Analysis Toolkit for the Multicellular Interactions (NATMI) [23], Connectome [24], iTALK [25], SingleCellSignalR [26], and CellPhoneDB [27]. LIANA provides an aggregate consensus rank of the ligand-receptor interactions from the five methods. To understand the potential downstream effects of ligand-receptor interactions produced by LIANA, we used NicheNet version 2.2.0 [28] to predict which ligands of sender cells are most likely to affect and regulate specific target genes in the receiver cells. These genes are searched for by NicheNet in receiver cells that may provide hypotheses concerning cell–cell communication. For our study, we defined ovarian cancer cells as sender cells and all CD8 + T cell subtypes as the receiver cells. In addition, we defined a ligand as expressed in the ovarian cancer cell if it was expressed in at least 40% of the cells, whereas we defined a receptor in CD8 + T cell subtypes as expressed if it was expressed in at least 20% of the cells. Further, we defined the gene set of interest that is likely to be influenced by cell–cell communication in our analysis. We included all genes that were differentially expressed between terminally exhausted CD8 + T cells and the precursor terminally exhausted CD8 + T cells. We focused on predicting target genes in terminally exhausted CD8 + T cells, as the state of exhaustion in CD8 + T cells is important for immunotherapy response. We used a sender-focused approach to include ligands expressed in ovarian cancer cells whose cognate receptors are expressed in terminally exhausted CD8 + T cells. The top 60 ligands identified by LIANA were further filtered to those included in NicheNet’s ligand-target matrix. The predicted ligands were ranked based on the area under the precision-recall curve (AUPR) between a ligand’s prior target predictions and the observed transcriptional response.

## Results

### Database setup and generation of integrated data

Four publicly available 10X genomics single-cell RNA-Seq datasets of twenty-three ovarian cancer patients selected from the GEO repository are summarized in Table 1. The quality control metrics applied during the pre-processing of all the datasets were visualized using violin plots (Supplementary Fig. 1A–D). A total of 199,540 cells and 38,629 genes were obtained before quality control, of which 153,226 cells and 35,013 genes remained after quality control. The total number of cells and genes in all the samples is summarized in Supplementary Table S1. In addition, the clinical data associated with the samples from dataset GSE173682, GSE154600, and GSE184880 is described in Supplementary Table S2. PCA plots were visualized for both merged and integrated data, which showed the removal of batch effects (Supplementary Fig. 2A). The UMAP plots of the merged dataset without batch correction showed the separation of clusters by samples, while the UMAP of the integrated dataset showed an even distribution of cells from each sample in each cluster (Supplementary Fig. 2B), verifying successful integration of the data.

### Cell type annotation reveals large T cell populations

**Fig. 2:**
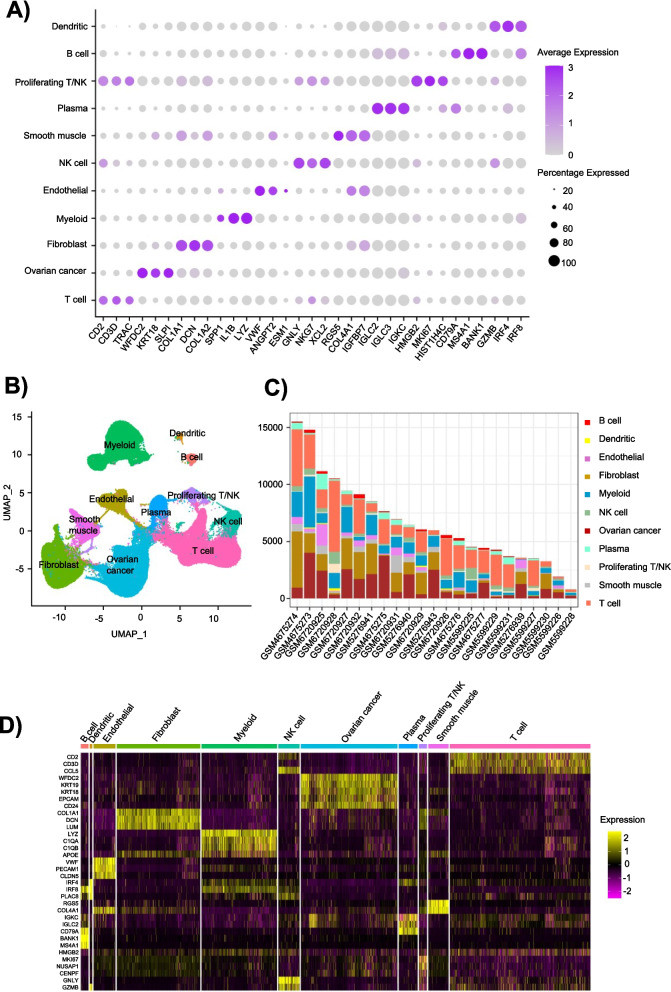
Overview of the cell type designation. **A** Dotplot shows top three marker genes used for cell type differentiation in at least one cluster. **B** Annotated clusters based on marker genes differentially expressed and from previous published studies shows 11 distinct cell types. **C** Distribution of cell types in each sample shows high number of T cell population. **D** Heatmap shows genes differentially expressed in each cell type

Clusters were annotated based on the expression of marker genes differentially expressed in different cell types (Supplementary Table S3) and based on markers used in previous studies (Table 2). The expression of the marker genes is highlighted in the dot plot (Fig. 2A). Altogether, eleven cell types were annotated, including B cells, T cells, fibroblasts, myeloid cells, ovarian cancer cells, endothelial cells, smooth muscle cells, plasma cells, dendritic cells, natural killer (NK) cells, and proliferating T/NK cells (Fig. 2B). The distribution of these major cell types across each sample (Fig. 2C) showed that the T cell populations were highest in the sample GSM6720928, followed by GSM4675274; however, these samples lack clinical data. Hence, the underlying reason for the high proportion of T cells in these samples remains unclear. The heatmap plot (Fig. 2D) also highlighted the different marker genes differentially expressed in each particular cell type.

### Pseudotime trajectory analysis reveals the transition states of CD8 + T cells

**Fig. 3:**
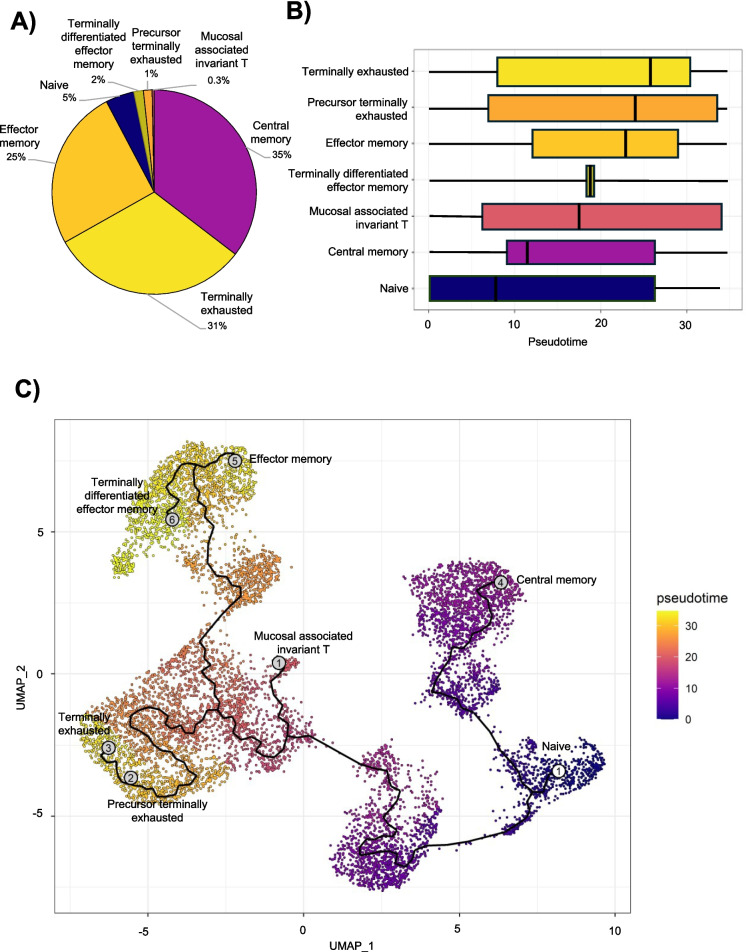
T-cell trajectory analysis. **A** Total number of CD8 + T cell subtypes identified using ProjectTILs with human CD8 + T cell reference shows central memory, effector memory, and terminally exhausted CD8 + T cells as the top 3 cell population **B** Boxplot shows the distribution of pseudotimes for each CD8 + T cell subtypes with terminally exhausted CD8 + T cells having the highest median pseudotime. **C** Pseudotime trajectory analysis of CD8 + T cell subtypes using Monocle3 shows the transition of naïve state to six different states

Our analysis of T cells using the human CD8 + TIL reference atlas (Supplementary Fig. 3A) resulted in 9,570 CD8 + T cells across all samples. The key marker genes of the reference CD8 + TIL cells overlapped with the query T cells (Supplementary Fig. 3B). The cells were classified as naive (CD8.NaiveLike), central memory (CD8.CM), effector memory (CD8.EM), terminally exhausted (CD8.TEX), terminally differentiated effector memory (CD8.TEMRA), precursor terminally exhausted (CD8.TPEX), and mucosal-associated invariant T (CD8.MAIT), with the proportion of central memory (35%), effector memory (31%), and terminally exhausted (25%) CD8 + T cells being higher across the samples (Fig. 3A). Almost each of the samples showed high proportions of central memory cells, while samples GSM6720928 and GSM5599227 showed the highest proportion of terminally exhausted cells (Supplementary Fig. 4A). The arrangement of the pseudotime values in the trajectory showed the median pseudotime for each of the CD8 + T cell subtypes (Fig. 3B). It was observed that the effector memory, precursor terminally exhausted, and terminally exhausted CD8 + T cells were in late differentiation stages with higher median pseudotime. Further, selecting memory or naïve cell marker genes (*KLF2*, *IL7R*) and exhausted cell marker genes (*GZMB, LAG3*) showed how these genes were changing expression in pseudotime along the trajectory (Supplementary Fig. 4B). The pseudotime trajectory UMAP plot (Fig. 3C) showed the different endpoints for each of the CD8 + T cell subtypes in pseudotime. The naive cells were found to be differentiating into central memory cells, which were then transitioning to precursor terminally exhausted and terminally exhausted states. The effector memory cells also exhibited higher pseudotime, suggesting the active functionality of CD8 + T cells.

### Potential interactions uncovered between ovarian cancer cells and terminally exhausted CD8 + T cells

**Fig. 4:**
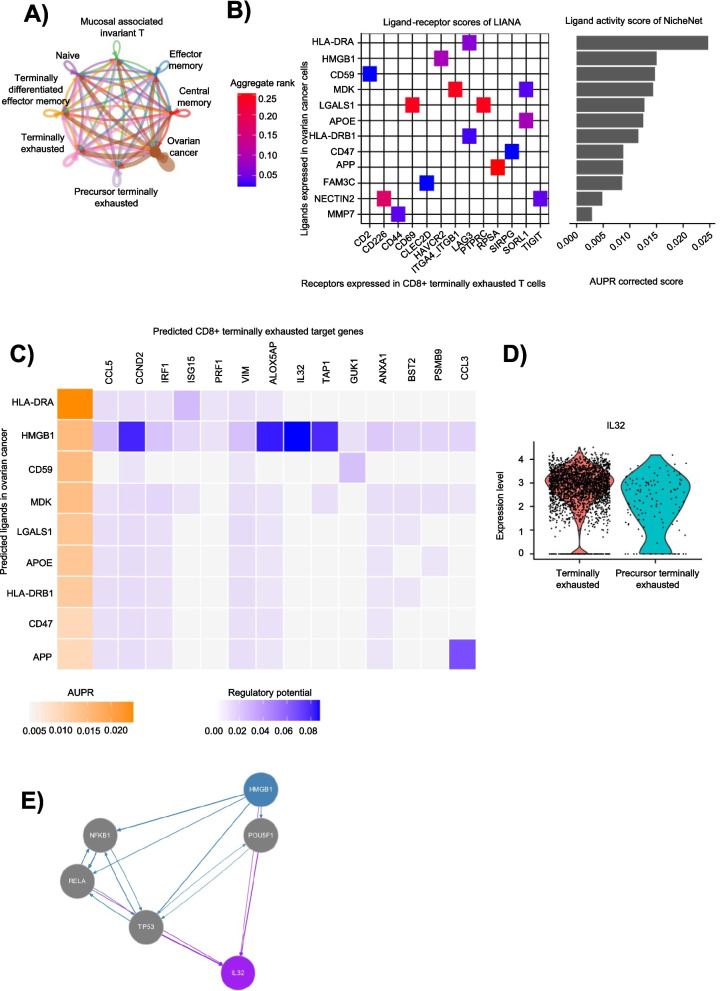
Cell–cell communication analysis. **A** CellChat predictions shows the highest number of interactions from ovarian cancer to CD8 + T cell subtypes. The thicker the lines, the more the number of interactions. **B** LIANA and NicheNet analysis shows *HMGB1*-*HACVR2* ligand-receptor interaction predicted by LIANA and the *HMGB1* ligand activity score predicted by NicheNet. **C** NicheNet predictions shows *HMGB1* in ovarian cancer cells with the highest ability to affect downstream target gene *IL32* expression in terminally exhausted CD8 + T cells. **D** Violin plot shows high expression of *IL32* in terminally exhausted CD8 + T cells compared to precursor terminally exhausted CD8 + T cells. **E** Network plot shows *NFKB1* as the most significant signaling mediator and *TP53* as the most significant transcriptional regulator involved in signaling from ligand *HMGB1* to target gene of interest *IL32*. The ligand node is indicated in blue, the target gene node in purple and nodes of signaling and transcriptional regulators in grey. Edges representing signaling interactions are colored blue, gene regulatory interactions in purple. The thicker the edge line, the more the weight of the represented interaction

CellChat analysis between ovarian cancer and CD8 + T cell subtypes resulted in 63 unique pathways and 207 ligand-receptor pairs for the secreted signaling communication group and seven unique pathways and 72 ligand-receptor pairs for the ECM-receptor signaling communication group. Based on the differentially over-expressed ligands and receptors, CellChat assigned the interactions with a *p*-value < 0.05 for each cell group (Supplementary Table S4). Ovarian cancer cells showed the highest number of interactions with effector memory, central memory, and terminally exhausted CD8 + T cells (Fig. 4A).

Cell–cell communication analysis using LIANA predicted ligand-receptor pairs based on the mean, median, and aggregate consensus ranks (Supplementary Table S5) between ovarian cancer cells and CD8 + T cell subtypes. Comparing CellChat and LIANA’s ligand-receptor interactions, we found 148 unique ligand-receptor pairs that were common between them (Supplementary Table S6). The top 12 ligands prioritized by LIANA were used to obtain the ligand activity predictions in NicheNet (Fig. 4B). Genes *HLA-DRA* and High Mobility Group Box 1 (*HMGB1*) showed higher NicheNet scores, indicating that they are most likely to have a strong influence on target genes. NicheNet prioritized ligands based on the presence of the target genes in the terminally exhausted CD8 + T cells (Supplementary Table S7). Genes in the gene set of interest, or active target genes that have the highest regulatory potential for each top-ranked ligand, were identified. We found that while *HLA-DRA* showed the highest ligand activity for target genes in terminally exhausted CD8 + T cells, *HMGB1* showed a high regulatory score for regulating the target genes *CCND2, ALOX5AP, TAP1*, and *IL32* (Fig. 4C). These target genes were identified based on the ligand-target regulatory potential scores between ligands of interest and their target genes. *IL32* showed a higher regulatory potential score compared to *CCND2, ALOX5AP*, and *TAP1* (Supplementary Table S8). These results indicate that *IL32* is likely influenced by *HMGB1* (Fig. 4B). We also observed high expression of *IL32* in terminally exhausted CD8 + T cells compared to precursor terminally CD8 + T cells (Fig. 4D). We further determined the weighted integrated signaling and gene regulatory networks to infer the most important signaling mediator and transcriptional regulator involved in signaling from *HMGB1* to *IL32* (Fig. 4E). The data sources supporting these interactions are in Supplementary Table S9. Interestingly, based on the edge weights of the networking plot, *TP53* was the most upstream of target gene *IL32* and most downstream of ligand *HMGB1*, making it the most significant transcriptional regulator regulating the expression of IL32. However, *HMGB1* to *NFKB1* was the most significant signaling axis in the predicted signaling network. These results indicate that *HMGB1* influenced *IL32* expression via *NFKB1* and *TP53*.

## Discussion

We found that while central memory and effector memory CD8 + T cells were abundant, a high proportion of terminally exhausted CD8 + T cells indicated an immunosuppressive tumor microenvironment. We also show that pseudotime analysis of CD8 + T cells revealed the early differentiation of CD8 + T cells into precursor terminally exhausted and terminally exhausted cell states. This result indicates that the CD8 + T cells are tumor specific. Their differentiation transitioned to an anergic and early dysfunctional state after encountering antigens in a non-inflammatory and non-stimulatory context [29].

Upon tumor antigen encounter, CD8 + naive cells are activated and then differentiated into memory cells: effector memory and central memory, that can progress further to exhausted cell state [30]. Exhausted CD8 + T cells also include a subset of progenitor exhausted cells that persist for the long term and show enriched signatures for cytokine production, survival, and memory [31]. Immunotherapy has been found to enhance the effectiveness of CD8 + T cells within the tumor microenvironment [32, 33]. Increased infiltration of CD8 + T cells showed a favorable prognosis and improved therapeutic efficacy in high-grade serous ovarian cancer [34]. Increased levels of CD8 + T cells improved survival and eliminated fibroblast-associated chemoresistance in high-grade serous patients by altering the metabolism of cysteine and glutathione [35]. Furthermore, the interaction of CD8 + T cells with tumor cells was shown to contribute to CD8 + T cell exhaustion in the ovarian cancer tumor microenvironment [36]. Therefore, understanding how tumors drive the exhaustion of CD8 + T cells is crucial for enhancing immunotherapy efficacy. Also, gene regulatory effects on the target gene expression in exhausted states of CD8 + T cells could contribute to a functional understanding of cellular communication in the tumor microenvironment.

Our findings revealed *HMGB1* as the key ligand expressed in ovarian cancer cells. *HMGB1* has multiple functions based on its location inside or outside the cell. It can act as a DNA chaperone, promote autophagy, or regulate inflammation and immune responses [37]. *HMGB1* plays a crucial role in tumor progression [38]. Inhibiting *HMGB1* was associated with anti-tumor effects in mouse tumor models [39]. Ovarian cancer patients with primary tumors exhibited high serum concentrations of *HMGB1*, making them a potential diagnostic biomarker [40]. Furthermore, the release of *HMGB1* was reported during the event of immunogenic cell death in ovarian carcinoma cells [41], highlighting the importance of targeting *HMGB1* to restore immunogenicity in ovarian cancer.

In our analysis, *HMGB1* influenced *IL32* expression in terminally exhausted CD8 + T cells. *IL32* is a pro-inflammatory cytokine associated with inflammation and cancer [42]. Cytokines such as interleukins are key mediators in cell–cell communication in the tumor microenvironment. These interleukins can be anti-tumorigenic [43] but also immune-suppressive [44]. Interleukins like *IL-2* were found to trigger CD8 + T cell exhaustion in the tumor microenvironment [45]. *IL32* and *ITGB3* crosstalk between cancer associated fibroblasts and breast cancer cells has been found to promote tumor cell invasion and metastasis [46]. An isoform of *IL32* was reported to be a crucial factor in tumor T-cell immune evasion and expansion of malignant T cells in cutaneous T-cell lymphoma [47].

Our results also highlighted *HMGB1* signaling *TP53*. *TP53* is the most frequently mutated gene in cancer [48] and is known for its role in regulating apoptosis, DNA repair, and cell cycle [49]. *TP53* also promotes immune escape and suppresses immune surveillance [50]. *TP53* has been found to be highly mutated in high-grade ovarian cancer [51] which suggests that *IL32* regulation and *TP53* together might promote an immunosuppressive tumor microenvironment. Additionally, in the predicted signaling network, *HMGB1* strongly interacted with *NFKB1* in terms of signaling, suggesting its role in stimulating the production of pro-inflammatory cytokines and chemokines [52]. Our results also show that *HAVCR2* (also known as *TIM-3*), a marker for exhausted CD8 + T cells, is the receptor for *HMGB1* in the ovarian cancer tumor microenvironment. This indicates that *HMGB1*-*HAVCR2* interactions might be involved in inhibiting *NFKB1* and contributing to the negative regulation of the tumor.

Several ligand-receptor interactions or pathways have been implicated in T-cell exhaustion across different cancers. The most well-known example is the *PD1*-*PDL1* axis, which contributes to T-cell inactivation and exhaustion in multiple cancer types [53]. Similarly, Galectin-9, a key ligand for *TIM3*, has been reported to induce the exhaustion of CD8 + tumor-infiltrating lymphocytes through the *TIM3*-Galectin9 pathway in diffuse large B-cell lymphoma [54]. In triple-negative breast cancer, the *CD155*-*TIGIT* signaling axis promoted CD8 + T cell exhaustion by modulating glucose metabolism [55] while in lung cancer, *STK31* induced exhaustion of CD8^+^ T cells by activating the *STAT3*/*IL-6* pathway [56]. Altogether, these findings illustrate that identifying novel signaling pathways and uncovering their role in T-cell exhaustion is an important basis for potential therapeutic strategies in cancer.

While there have been several therapeutic strategies targeting *HMGB1* in various cancer types [39, 57], there are currently no *IL32* targeted therapies. Also, the precise role of *IL32* in terminally exhausted CD8 + T cells in the tumor microenvironment of ovarian cancer remains unclear. Current immunotherapy approaches like anti-PD1 and anti-CTLA4 for solid tumors depend on a sufficient population of intra-tumoral T cells to enhance the T cell anti-tumor activity. Combination of these approaches with cytokine immunotherapy may be a solution to this limitation, as cytokines have the natural ability to trigger tumor antigen expression. Clinical trials have also highlighted the efficacy of cytokine-based therapies. For instance, IL2 was approved for metastatic renal cell carcinoma and metastatic melanoma to enhance T-cell responses [58], while IL15 was approved for the treatment of bladder cancer [59]. In addition, several pre-clinical studies conducted also showed the potential of interleukins like IL4 [60] and IL12 [61] for improving clinical response. Despite success, the heterogeneity of tumors and the complexity of the tumor microenvironment remain a major challenge.

There are a few limitations in our study. Our sample size was relatively small, and incorporating more patient samples could help to better understand cell–cell communication and their regulatory effect. Nevertheless, about two hundred thousand cells were evaluated in our study. Second, the cell–cell communication methods are not based on a direct measurement of proteins. Therefore, future studies using multiple levels of data are warranted to confirm whether *IL32* expression is a driver of exhaustion or merely a downstream marker of exhaustion.

In conclusion, our study revealed cellular heterogeneity of CD8 + T cells in the tumor microenvironment of ovarian cancer. The transition of CD8 + T cells in the developmental trajectory was investigated, and the most significant ligand in ovarian cancer cells and target gene in terminally exhausted CD8 + T cells were identified. Overall, our study highlights that *HMGB1* influencing *IL32* may represent a promising therapeutic strategy to reverse T cell exhaustion and enhance anti-tumor immunity. Future confirmation of *HMGB1* and *IL32* expression through western blot or immunohistochemistry and functional assessment through in vivo or in vitro experiments using activators or inhibitors for *HMGB1* or *IL32* would validate whether targeting *the HMGB1–IL32* axis could restore T cell functionality or delay T cell exhaustion. Finally, we also hypothesize that uncovering the exact role of *IL32* in tumor immunity may pave the way for new effective therapies.

## Supplementary Information

Below is the link to the electronic supplementary material.

ESM 1 (XLSX 1.44 MB)

ESM 1 (PDF 1.39 MB)

## Data Availability

The datasets analyzed in the study are available in the GEO repository using their respective identifiers or accessible via the indicated PubMed identifiers. The R codes used in the study will be made available on request.
