# Supplementary material for: Targeting the HMGB1-IL32 pathway to alleviate T cell exhaustion in epithelial ovarian cancer
Source: GeroScience. 2025 Nov 15;48(3):3335–50. doi: 10.1007/s11357-025-01963-5 (PMC13356217; doi:10.1007/s11357-025-01963-5)
Supplement: Supplementary file 2 — (PDF 1.39 MB) [file 11357_2025_1963_MOESM2_ESM.pdf]

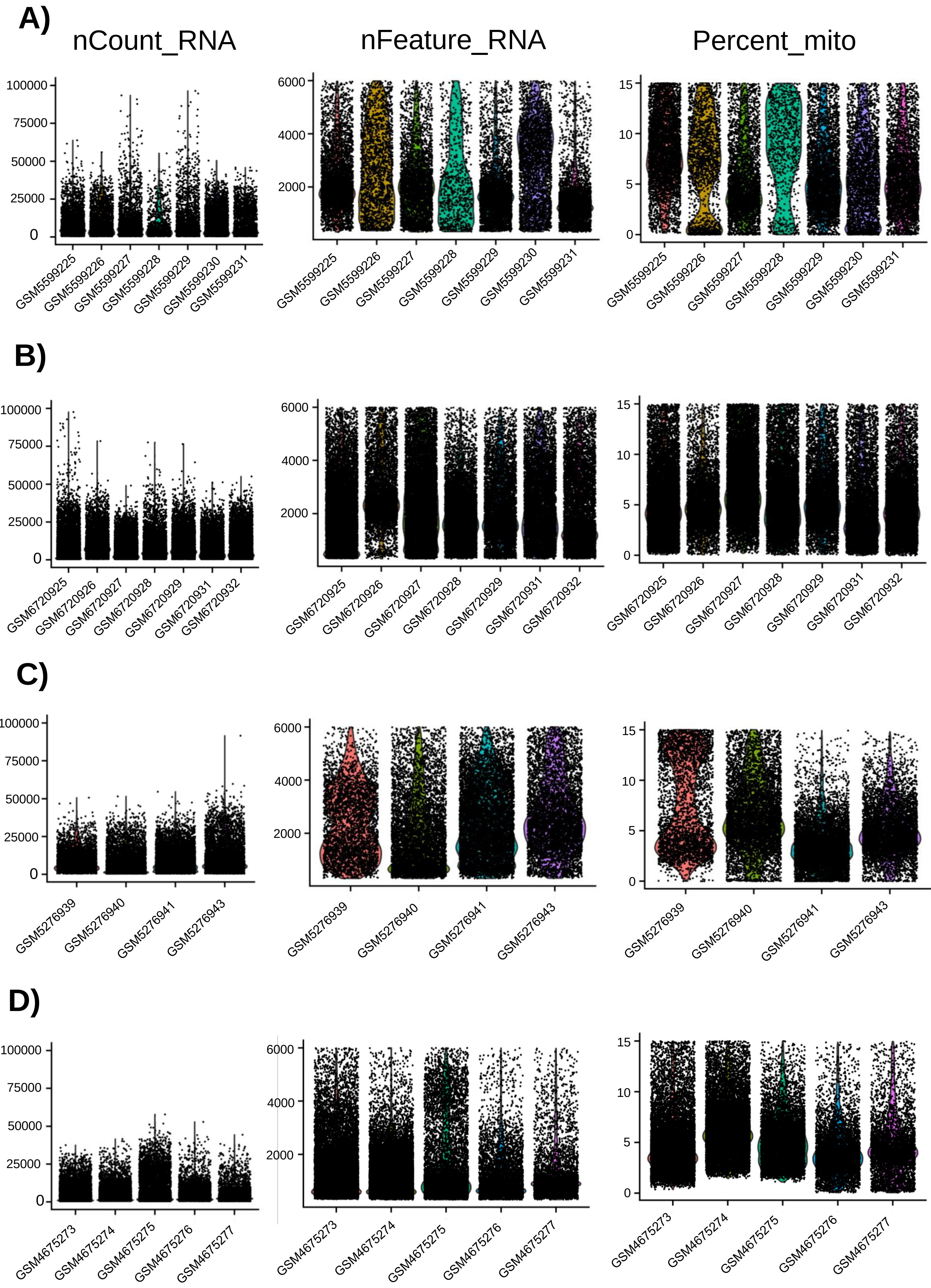


**Supplementary Figure 1.** Quality control plots samples from datasets GSE184880 **(A)**, GSE217517 **(B)**, GSE173682 **(C),** GSE154600 **(D)**.

A)

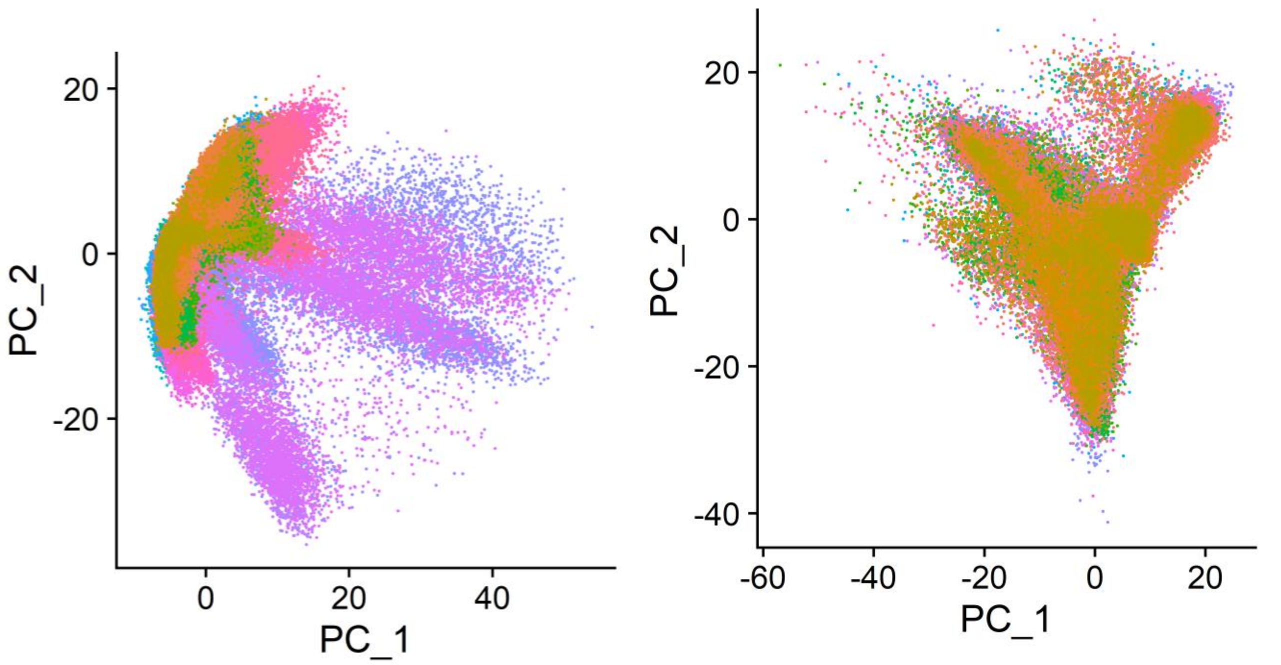


B)

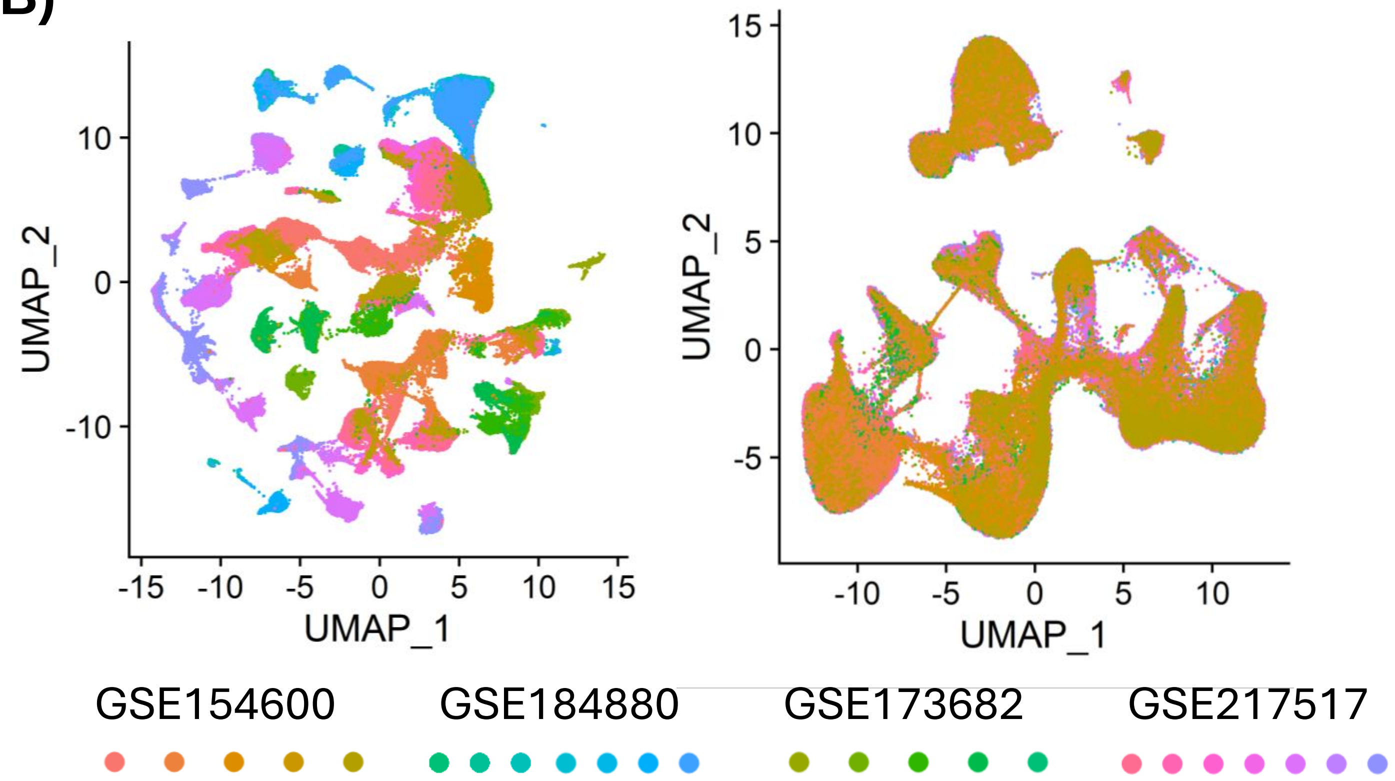


**Supplementary Figure 2** PCA plots before batch correction (left) and after batch correction (right) **(A)** UMAP plots before batch correction (left) and after batch correction (right) **(B)**

A)

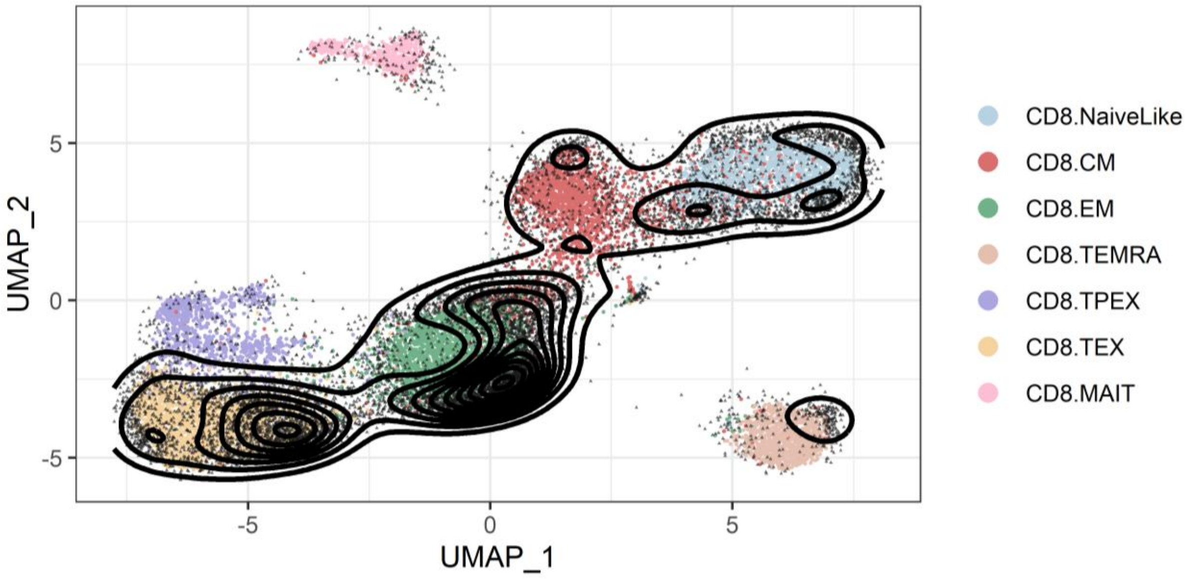


B)

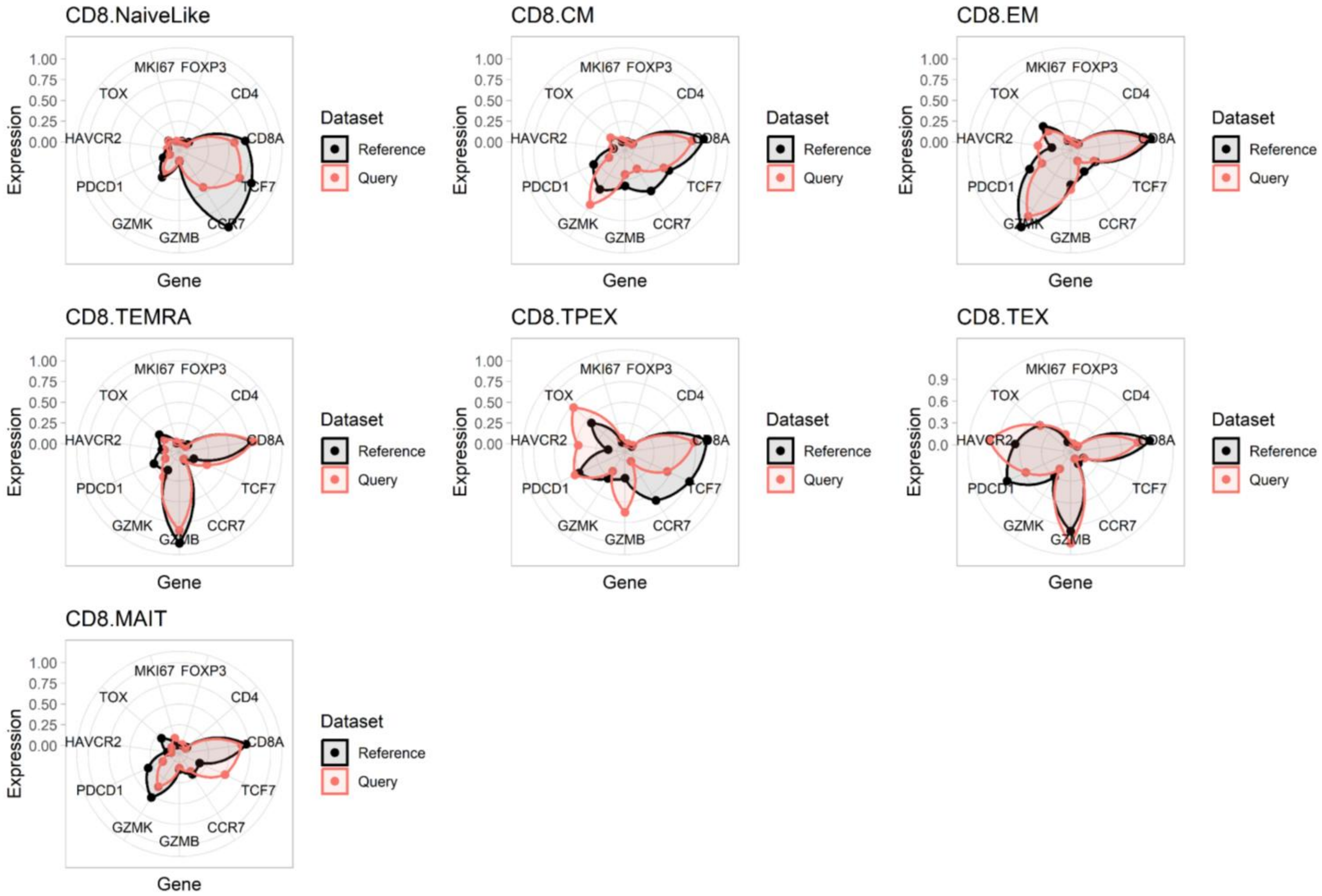


**Supplementary Figure 3.** Query T cells (black circles) projected on human CD8+ TIL atlas reference map using ProjectTILs **(A)** Radar plots showing gene expression signature of query cells (red) and human reference CD8+ TIL (black) of the seven CD8+ T cell subtypes **(B)**

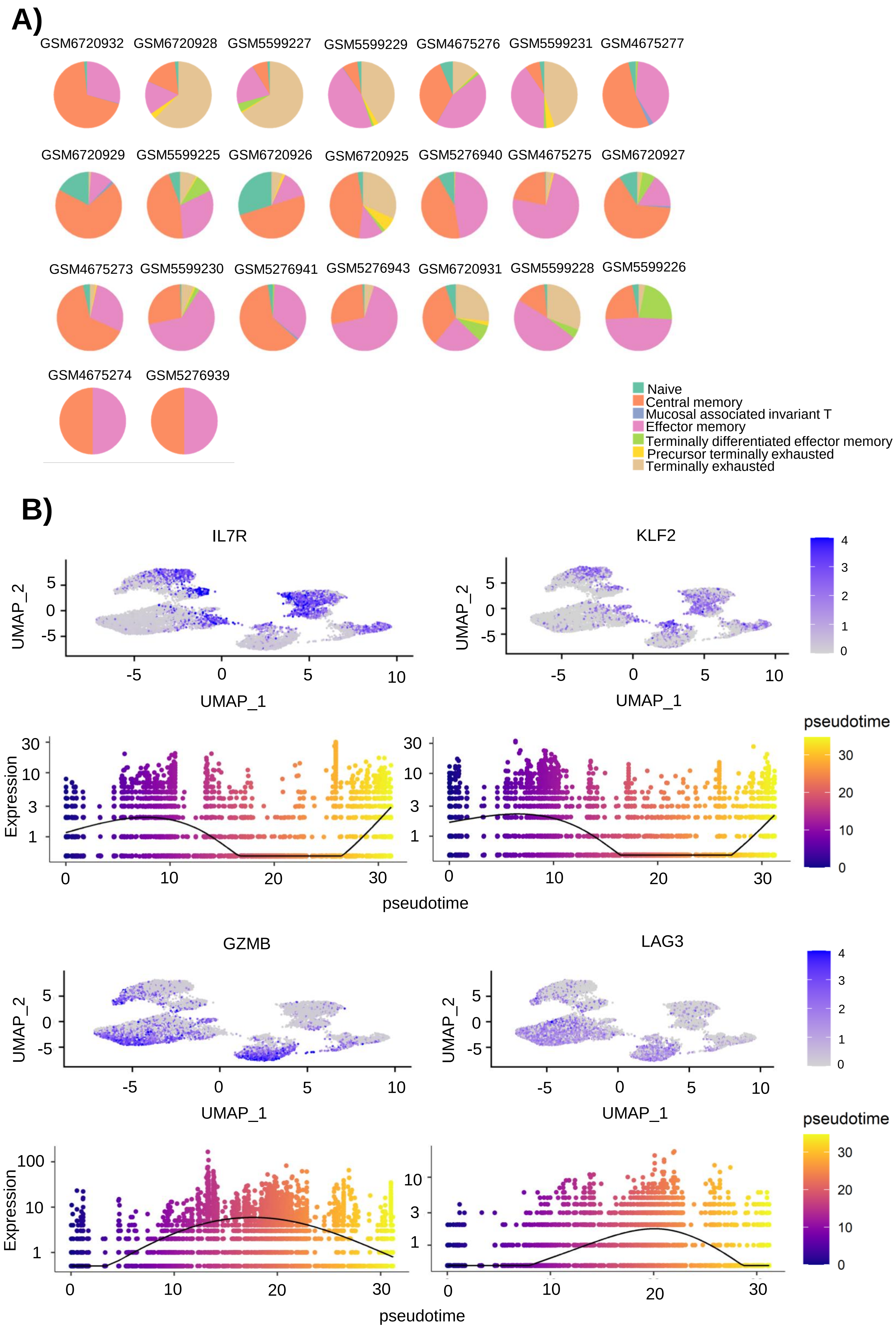


**Supplementary Figure 4.** Distribution of CD8+T cell subtypes in each sample **(A)** Naïve, memory and exhausted genes changing expression in pseudotime **(B)**
